# Supplementary figures and images for: A novel panel of Drosophila TAFAZZIN mutants in distinct genetic backgrounds as a resource for therapeutic testing
Source: PLoS One. 2023 Sep 27;18(9):e0286380. doi: 10.1371/journal.pone.0286380 (PMC10529581; doi:10.1371/journal.pone.0286380)

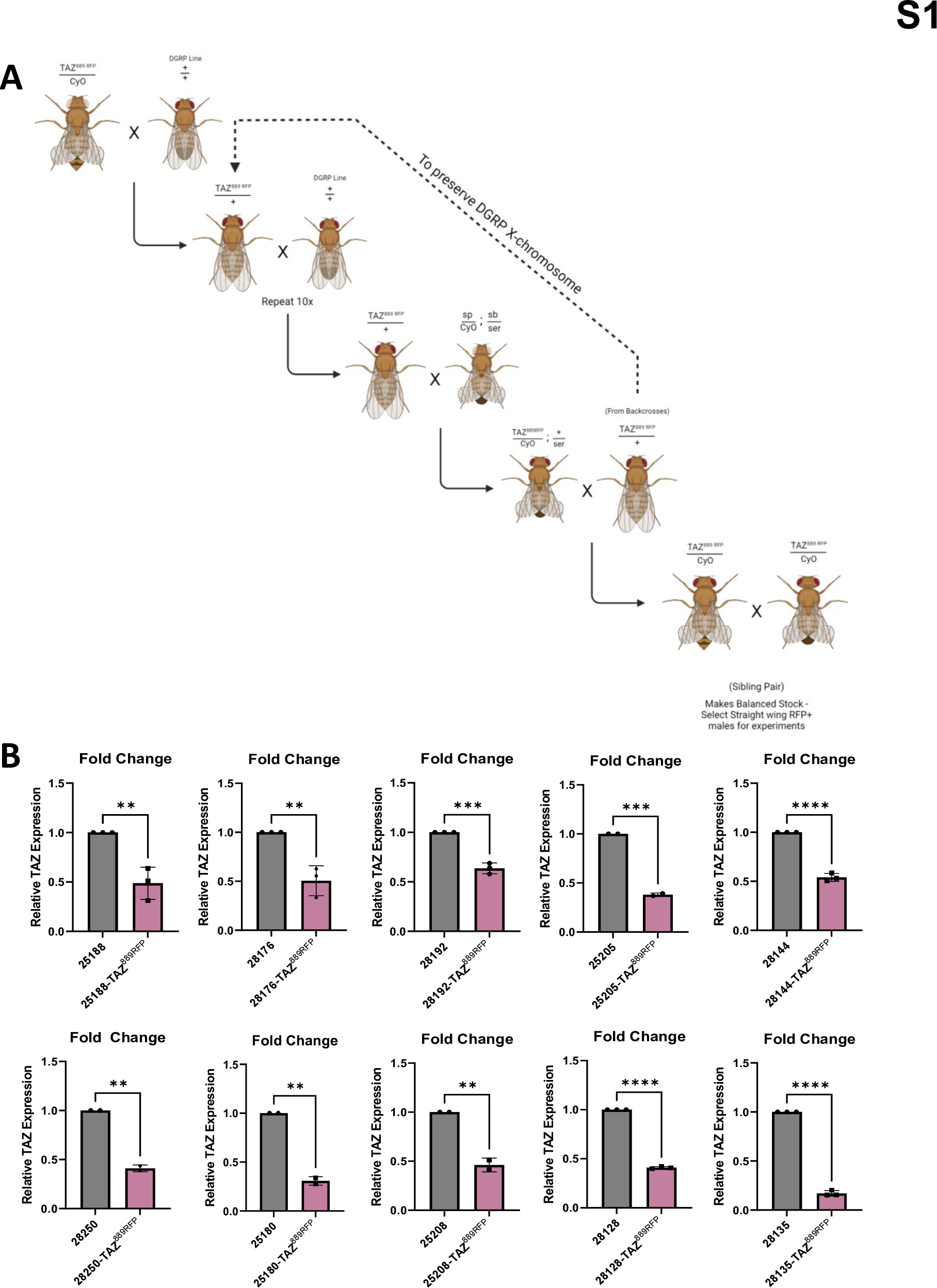

Supplement: S1 Fig — (A) Schematic representation of backcrossing scheme used to generate the 10 new DGRP-TAZ mutant lines. Created with BioRender.com (B) PCR results confirming reduced TAZ expression in each of the 10 backcrossed DGRP background lines, two tailed, unpaired Student’s t-test. *P<0.05, **P<0.01, ***P<0.001, ****P<0.0001. (TIF) [file pone.0286380.s001.tif]

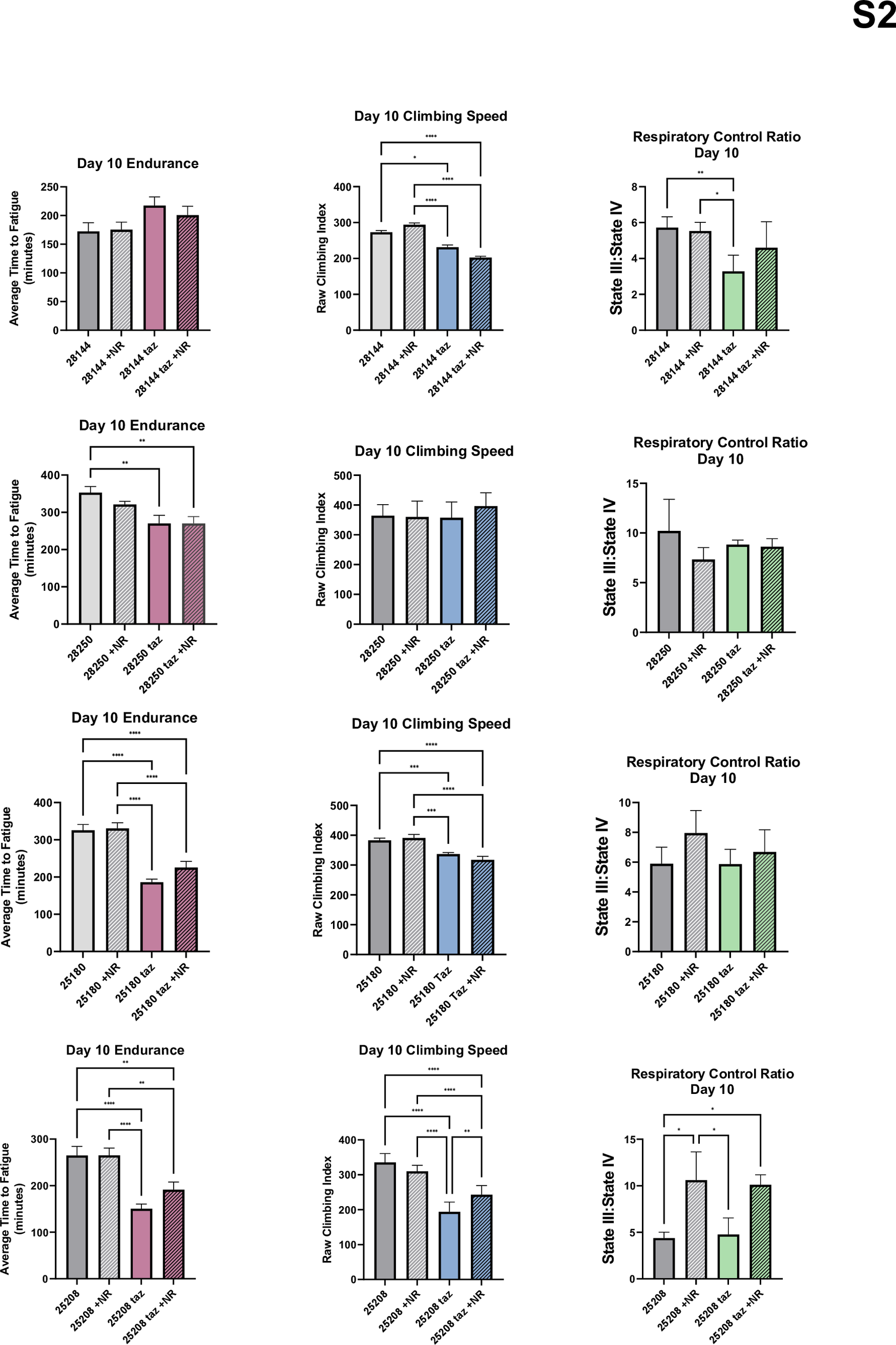

Supplement: S2 Fig — Day 10 average endurance (red, n = 10 vials, 200 flies), climbing speed (blue, n = 100 flies) and mitochondrial RCR (green, n = 3 biological replicates) with NR supplementation. Two-way ANOVA + Tukey. *P<0.05, **P<0.01, ***P<0.001, ****P<0.0001. Despite the large separation of average RCR, the benefit provided to mitochondrial RCR by NR supplementation related to line 25208 showed a large degree of variation across repetitions in 2 of the 4 cohorts (25208 +NR and 25208 TAZ), therefore resulting in modest p-values or non-significant results: 25208 vs 25208 +NR: *P = 0.033, 25208 vs 25208 TAZ: P = 0.99, 25208 vs 25208 TAZ +NR: *P = 0.046, 25208 +NR vs 25208 TAZ: *P = 0.044, 25208 +NR vs 25208 TAZ +NR: P = 0.98, 25208 TAZ vs 25208 TAZ +NR: P = 0.062. (ZIP) [file pone.0286380.s002.zip › S2_Fig (2).tif]

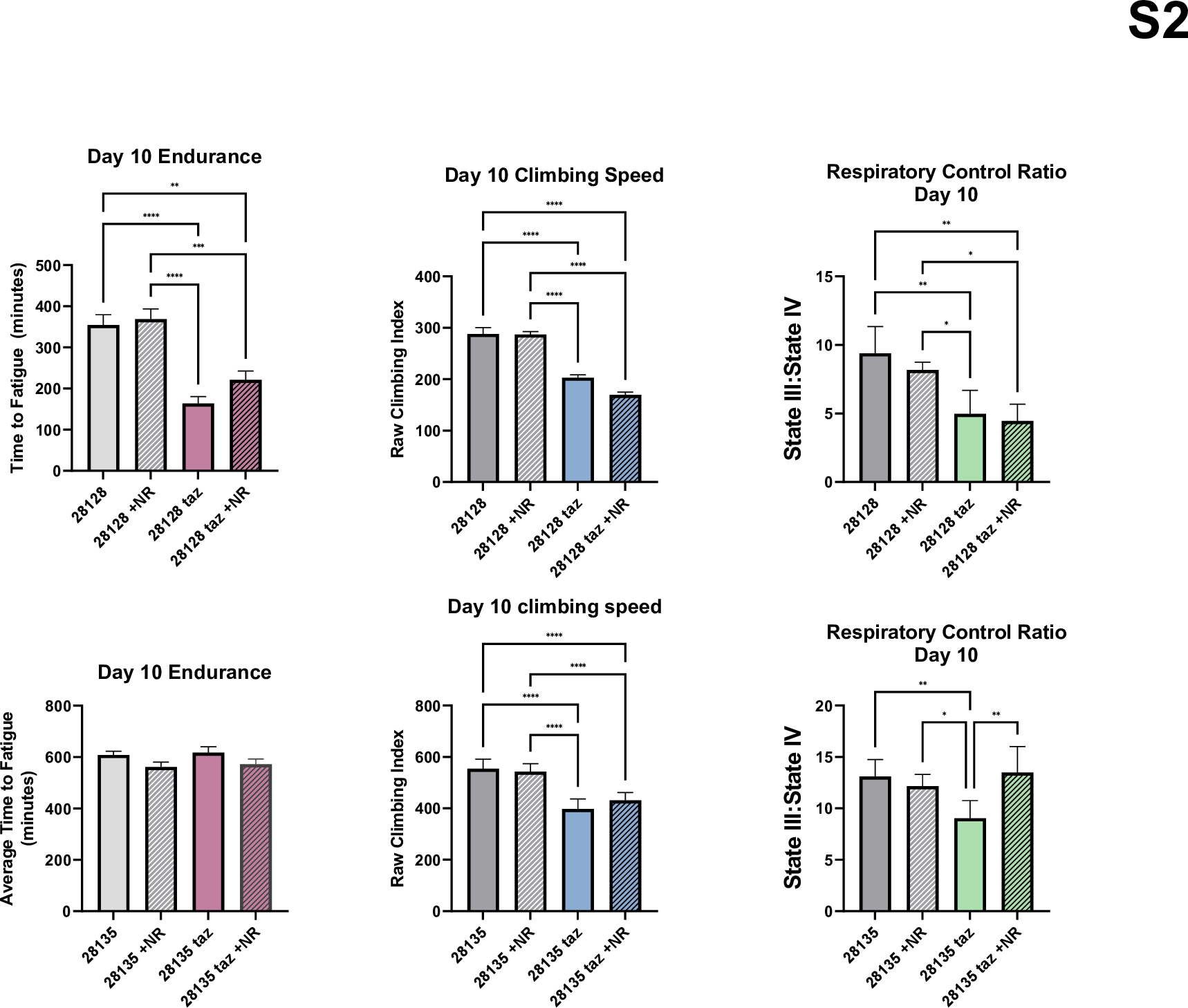

Supplement: S2 Fig — Day 10 average endurance (red, n = 10 vials, 200 flies), climbing speed (blue, n = 100 flies) and mitochondrial RCR (green, n = 3 biological replicates) with NR supplementation. Two-way ANOVA + Tukey. *P<0.05, **P<0.01, ***P<0.001, ****P<0.0001. Despite the large separation of average RCR, the benefit provided to mitochondrial RCR by NR supplementation related to line 25208 showed a large degree of variation across repetitions in 2 of the 4 cohorts (25208 +NR and 25208 TAZ), therefore resulting in modest p-values or non-significant results: 25208 vs 25208 +NR: *P = 0.033, 25208 vs 25208 TAZ: P = 0.99, 25208 vs 25208 TAZ +NR: *P = 0.046, 25208 +NR vs 25208 TAZ: *P = 0.044, 25208 +NR vs 25208 TAZ +NR: P = 0.98, 25208 TAZ vs 25208 TAZ +NR: P = 0.062. (ZIP) [file pone.0286380.s002.zip › S2_Fig (3).tif]

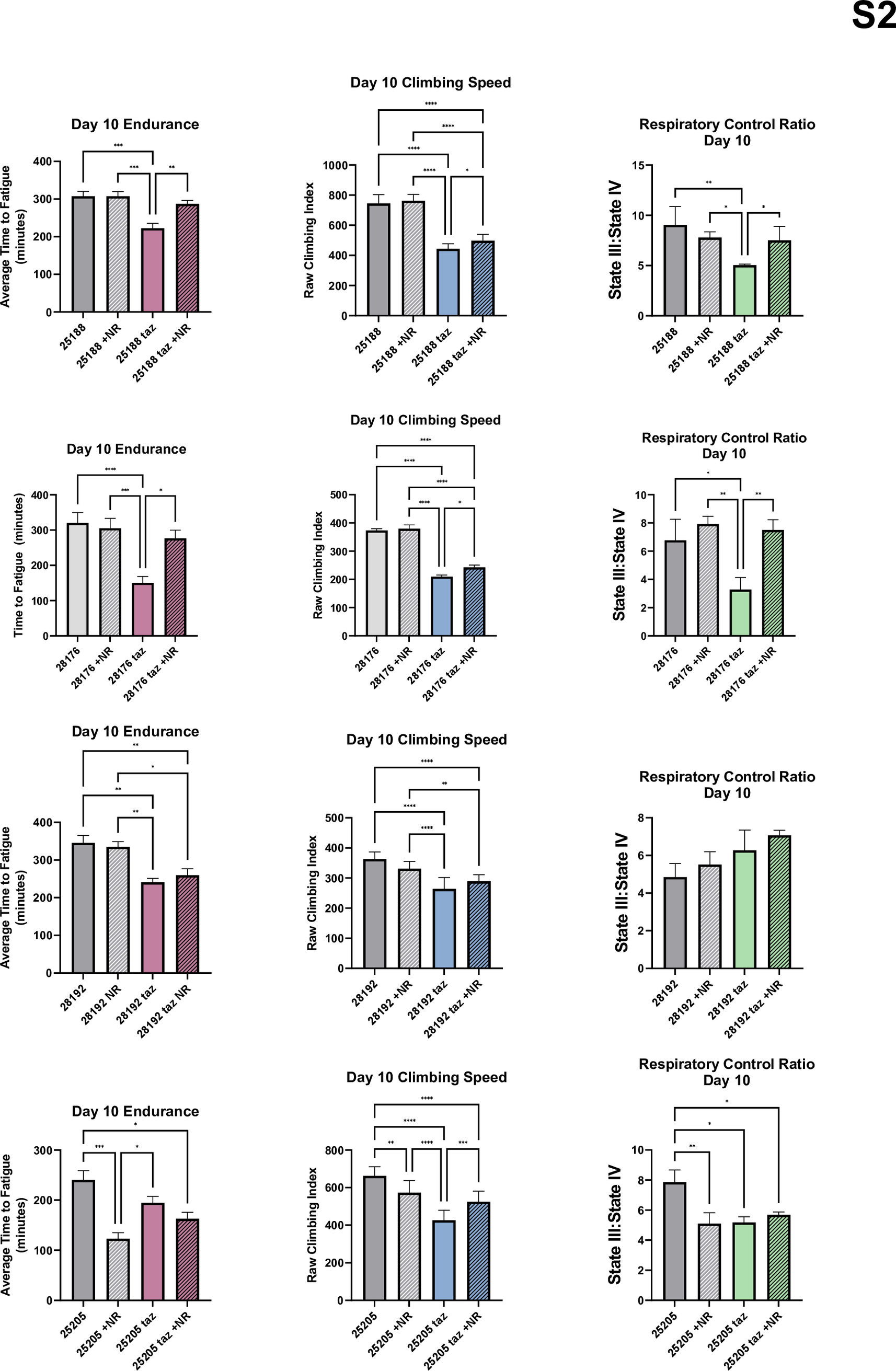

Supplement: S2 Fig — Day 10 average endurance (red, n = 10 vials, 200 flies), climbing speed (blue, n = 100 flies) and mitochondrial RCR (green, n = 3 biological replicates) with NR supplementation. Two-way ANOVA + Tukey. *P<0.05, **P<0.01, ***P<0.001, ****P<0.0001. Despite the large separation of average RCR, the benefit provided to mitochondrial RCR by NR supplementation related to line 25208 showed a large degree of variation across repetitions in 2 of the 4 cohorts (25208 +NR and 25208 TAZ), therefore resulting in modest p-values or non-significant results: 25208 vs 25208 +NR: *P = 0.033, 25208 vs 25208 TAZ: P = 0.99, 25208 vs 25208 TAZ +NR: *P = 0.046, 25208 +NR vs 25208 TAZ: *P = 0.044, 25208 +NR vs 25208 TAZ +NR: P = 0.98, 25208 TAZ vs 25208 TAZ +NR: P = 0.062. (ZIP) [file pone.0286380.s002.zip › S2_Fig.tif]

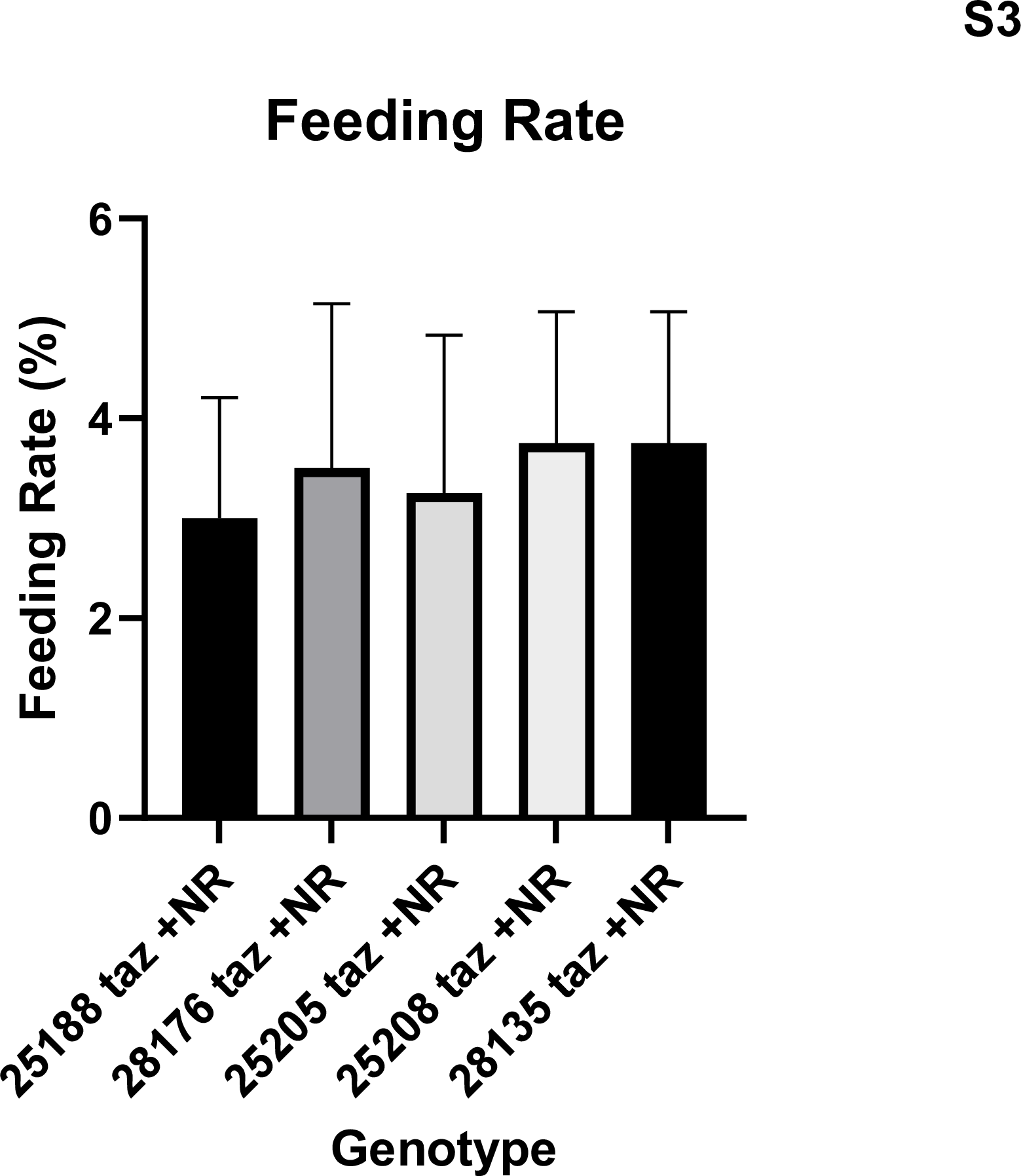

Supplement: S3 Fig — Lines included in this figure gained improvements in either endurance, climbing speed or mitochondrial RCR after 10 days of NR feeding. No significant difference was seen in feeding rates between these lines. (n = 10 vials, 100 flies) One-Way ANOVA, P = 0.7186. (TIF) [file pone.0286380.s003.tif]
