# Supplementary material for: A novel panel of Drosophila TAFAZZIN mutants in distinct genetic backgrounds as a resource for therapeutic testing
Source: PLoS One. 2023 Sep 27;18(9):e0286380. doi: 10.1371/journal.pone.0286380 (PMC10529581; doi:10.1371/journal.pone.0286380)
Supplement: Attachment — Submitted filename: Response letter.docx [file pone.0286380.s004.docx]

We thank the reviewers for their comments, as they have greatly improved the manuscript. Detailed descriptions of changes are outlined below:

Reviewer 1:

We agree with the reviewer that the number of lines in this study is not sufficient to perform GWAS analysis or identify specific modifier loci. However, this was not our purpose in the study. Rather, our goal was to generate a novel resource to facilitate rapid testing of potential interventions in a wide range of genetic backgrounds. In this way, potential therapies can be vetted early in the process through inexpensive fly trials before human testing. Pre-testing in these carefully generated and curated fly lines will allow potential therapies to be sorted into categories based on whether they are widely effective or are more effective in particular backgrounds. We feel this resource will be a significant help to the Barth research community in the future, whereas other strategies will need to be employed to identify specific modifier loci. The generation and testing of such a novel resource seems to us to be well within the mission of the PLoS ONE journal, and as such, we feel strongly that the manuscript should be considered for publication therein.

We have edited the text to clarify and emphasize the use of this study as a validation of a novel resource for BTHS researchers, and not as a means to actually identify modifiers of disease. (Lines: 93-95, 217-218, 239, 255-256, 263-264).

Reviewer 2:

- Introduction: please provide more details about the kind of Tafazzin mutations in Barth Syndrome and available fly models.

Please see Lines 39-43 and 72-77: Text outlining current Drosophila models of barth syndrome as well as literature on known mutations and their characterizations has been added.

- Lines 119-120 and 179-181: please provide data or reference of publication demonstrating that fly endurance and climbing involve different energetic requirements (i.e., sustained vs brief), otherwise remove these sentences or move them to the discussion section.

We have moved the referred to text into the discussion as suggested (Lines 206-212). We also clarify in our Methods section that speed is measured across 2 seconds (line 300), whereas endurance is measured until exhaustion (line 312), which typically requires hundreds of minutes (see Figures 1, 2 and supplemental).

- Lines 177-179: please correct references to Fig.3 panels.

Call outs to Figure 3 (now Lines 185 and 187) have been edited to match Figure 3 legend, and panel arrangement has been modified to correspond with those changes.

- Fig.3: please annotate fly lines in the dot plot.

Each data point has been labeled with the corresponding fly line in the revised Figure 3. We thank the reviewer for this suggestion, as it improves the figure substantially.

- In the Experimental procedures section, please detail how the “raw climbing index” was calculated.

Lines 303-308: Text has been added in Methods to describe the calculation of raw climbing index for a given genotype. Generally, the term raw we use to mean the data for each group is not normalized to its starting point. In cases where data is normalized, we include the word “normalized” in the label.

- In the Experimental procedures section, please include detailed description of PCR assay and sequences of the primers used in Fig.S1.

We thank the reviewer for catching this oversight. Description now appears in lines 281-295 in experimental procedures section.

- Fig.S2: please provide statistics for line 25208 mitochondrial respiration rescue by NR.

The large variation in RCR values across repetitions is the reason for the visibly large separation of cohorts being associated with a modest p-value for this line. Along with the statistical tests completed, the exact p-value for this line and a short description have been added to the figure legend for Figure S2 (Lines 466-471).
